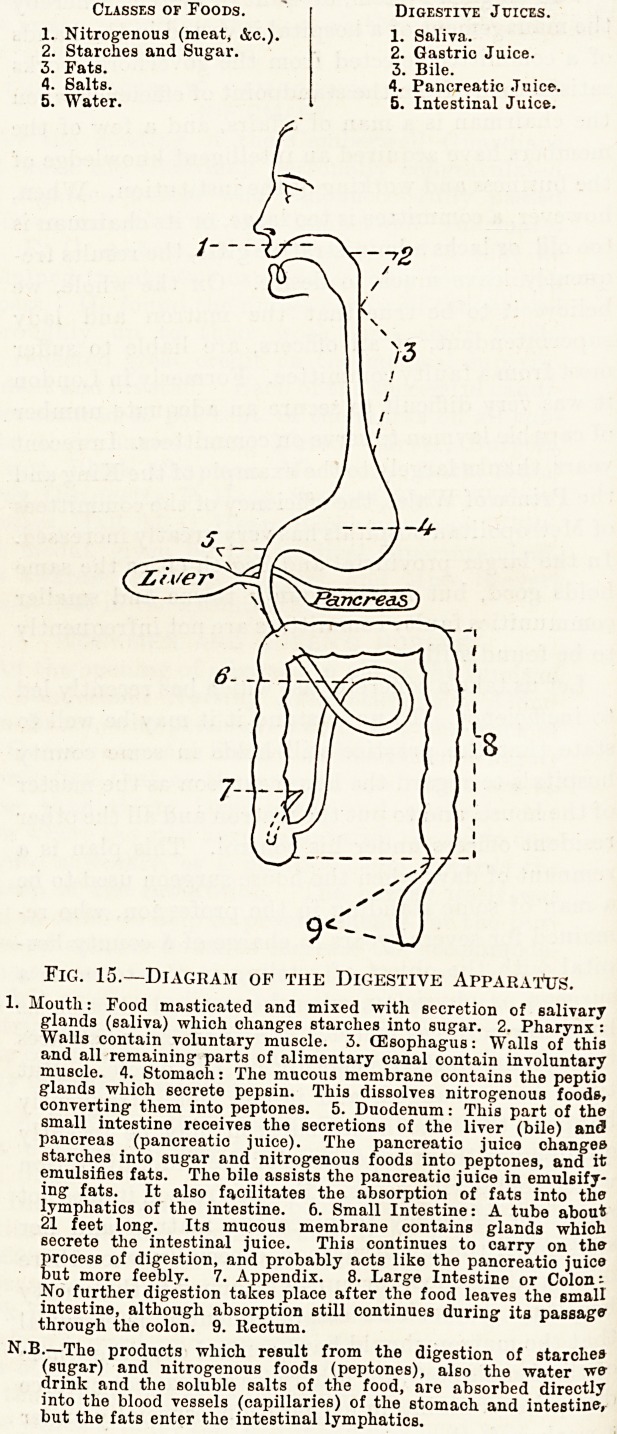# "The Hospital" Nursing Section

**Published:** 1906-05-19

**Authors:** 


### The Hospital.

### Nursing Section.

Contributions for "The Hospital," should be addressed to the Editor, "The Hospital" Nursing Section, 28 & 29 Southampton Street, Strand, London, W.C.

No. 1,025.—Vol. XL. SATURDAY, MAY 19, 1906.

### Notes on News from the Nursing World.

### MISS NIGHTINGALE'S BIRTHDAY.

On Tuesday Miss Florence Nightingale completed her eighty-sixth year, and, as usual on her birthday, she was the recipient of many congratulations from far and near. As on former occasions, most of the newspapers made a mistake in the date, and announced that the anniversary was celebrated on Saturday. Miss Nightingale, we are glad to say, suffers in no way from failure of mental powers, and continues to be able to take a keen interest in nursing matters.

### ENGLISH NURSES IN BELGIUM.

We are now in a position to sum up the prospects of openings for English nurses in Belgium, and it is quite clear that Brussels is the only place where there is any real demand for them. With regard to the provinces, we are informed by a well-known English medical man at Spa that it occasionally happens that he wants a nurse once or twice during the season from June to October, but he could not regularly employ one. The needs of the Belgian doctors are supplied by a Belgian religious order, which possesses an almshouse at Spa. At Bruges, although there is a large English colony, there is no resident English medical man, and in the event of an English nurse being asked for, her services could be easily secured by a wire to the capital, only a short distance away. Concerning the latter, our statement last week that there is an opening for a couple of English nurses in Brussels has been further confirmed. There are, however, a few conditions to be observed in addition to these which we have already specified. An acquaintance with another language, as well as a thorough knowledge of French, is very desirable. Maternity training is an absolute necessity. Roman Catholic nurses stand a better chance than Protestants of engagements amongst the Belgians, at any rate; and, to quote the words of our principal informant, "Nurses who would have to rely altogether upon the *clientèle* of the English community in Brussels, or Belgium, had better keep away." He also recommends that any English nurse anxious to settle in Belgium should send her photograph, full details as to her training, age, experience, requirements as to fees, and copies of testimonials, to the Lady Superintendent of the Victoria Institute, Rue de Vienne, Brussels; and as the city is comparatively empty until October, there is ample time to fully consider the matter before concluding matters. As to the establishment of a nursing home, we are emphatically assured that a substantial capital would be required to make it pay, because since the expulsion of the nursing sisterhoods from France a large number have migrated to Belgium and started nursing institutions, some on a very grand scale. Moreover, there are operating surgeons who prefer to have their patients nursed under their care.

### CENSURE OF A NURSE BY A JURY.

In returning a verdict of manslaughter on Friday against Dr. G. R. Adcock, the jury passed "the strongest censure possible" upon four other persons, including Miss Edith Jones, of Southfleet, a trained nurse, who attended the late Major Whyte during the illness which resulted in his death. Whatever may be the result of the charge against Dr. Adcock, it is satisfactory that a person who, in her evidence, stated that she had had extensive hospital experience, but that she attributed more importance to prayer than to nursing, should be severely condemned. There is no room in the nursing world for women who believe in the practice of faith-healing as a substitute for medical treatment.

### THE PROVOCATIONS OF MENTAL NURSES.

At Brentwood last week a former attendant at Essex Lunatic Asylum was fined £2 and costs for striking and ill-treating a patient. The defendant having pleaded guilty, evidence was given showing that she struck a patient on the side of the head with the back of a hair-brush. The patient had been very tiresome that morning, and in addition to knocking off one nurse's cap and striking another, had started fighting with a fellow patient. The medical superintendent subsequently said that the attendants often had had to endure great provocation, and that "it would take angels from heaven to keep their tempers." The nurse then stated that she acted in self-defence, the blow being given with the bristles. Nevertheless, the blow should not have been given, and a person who cannot keep her temper even under very severe provocation, is not well fitted for the work of nursing the mentally afflicted.

### FOURTEEN YORKSHIRE WORKHOUSES WITHOUT A NIGHT NURSE.

In his official return for 1905, Mr. P. H. Bagenal, Local Government Board inspector for the East and West Ridings of Yorkshire, states that the number of sick on December 31, 1905, in Poor-law Institutions was 5,157, and the number of nurses, day and night, 437, as well as 104 pauper attendants. The number of patients to each nurse averaged 11.80, this figure differing little from the return for December 31, 1904, which gave the average number of patients to each nurse as 11.95. In summing up,

Mr. Bagenal observes that the facts recorded show that the Guardians of the Poor in Yorkshire have not been neglectful of the general order issued by the Local Government Board in 1897 dealing with "the nursing of the sick in workhouses." But he adds: "The only weak point in the return is that in 14 workhouses it has not yet been found possible to provide a night nurse." This, however, is a very weak point, and strong efforts should be exerted without delay in order to ensure for the sick in these 14 institutions the attention which they imperatively need. No workhouse with sick wards should be without a night nurse.

### IMPERIAL MILITARY NURSING SERVICE.

We are officially informed that Nurses A. R. Sibbald, I. J. Egerton, V. L. Batteson, F. E. Manfield, and C. V. S. Johnson have been appointed staff nurses in Queen Alexandra's Imperial Military Nursing Service. Miss K. M. Hewetson, sister, has been transferred to the Royal Victoria Hospital, Netley, from the Royal Military College, Sandhurst, and Miss S. K. Bills, sister, to the Military Hospital, Curragh, from the Queen Alexandra Military Hospital, Millbank. Miss C. V. S. Johnson, staff nurse, has been posted to the Cambridge Hospital, Aldershot, on her appointment; and Miss F. E. Manfield, staff nurse, to the Royal Victoria Hospital, Netley. Miss O. M. Griffin has resigned her appointment as staff nurse.

### THE NURSES OF ST. GEORGE'S HOSPITAL.

The annual entertainment for the nursing staff of St. George's Hospital took place on Thursday and Friday last week. It was held each evening in the board-room of the hospital and was crowded on both occasions. The Treasurer and several members of the staff attended, and each nurse was allowed to bring one friend. Princess Victoria of Schleswig-Holstein kindly arranged the programmes and was present each evening, a bouquet being presented to the Princess at the close of the performance on the second evening by Sisters Horne and McIndoe, the senior sisters of the hospital. There was some excellent music and an admirable selection of songs was given, the performance each time concluding with the representation of the one-act comedy, "A Marriage has been Arranged." It was signified on the programmes that no encores could be accepted.

### THE ROYAL NATIONAL PENSION FUND.

On the 9th instant Mr. Louis Dick, the Secretary of the Royal National Pension Fund for Nurses, explained the advantages of joining the Fund to a meeting of nurses held at the General Hospital, Wolverhampton. Mr. Neil, the house governor, was in the chair, and in addition to district nurses from Stafford, Darlaston, Willenhall, and Bolton, the following matrons attended with their nurses: Miss Hannath, General Hospital; Miss Connoll, Eye Infirmary; Miss Mulligan, Fever Hospital; Miss Carter, Union Infirmary, Wolverhampton; Miss Parsons, Guest Hospital, Dudley; Miss Mutler, Burton Hospital; Miss Davis, Walsall Hospital; Miss Sharp, Rugeley Hospital; Miss Loveys, Queen Victoria Nurses' Institute, Wolverhampton; Miss Travis, Royal Orphanage, Wolverhampton; Miss Hunt and Miss Smith, West Bromwich Nurses' Home. The meeting proved very successful, and warm thanks were accorded to Miss Hannath for her hospitality and public spirit in calling the meeting.

### THIRTY-EIGHT YEARS' SERVICE.

An exceptionally interesting ceremony took place in the Norfolk and Norwich Hospital a few days ago, the occasion being the presentation of a number of gifts to Miss S. A. Bessey on her retirement, after 38 years' service, from the nursing staff of the institution. Miss Bessey entered the hospital in 1868 and has been nurse and subsequently sister ever since. She has necessarily a more intimate knowledge of its inner working than any living person, and when at last she concluded that her period of service should come to an end, the Board of Management decided both to grant her a small pension and to offer her a special gift in recognition of her life-work, which has been performed throughout to the satisfaction of the medical and surgical staff, as well as of successive matrons. This gift consisted of a purse containing 55 sovereigns, and it was presented to her in the name of the very numerous subscribers by Sir Charles Gilman, whose speech may be summarised in the words: "There was no question that everyone assembled greatly respected Sister Bessey." The honorary medical and surgical staff gave Miss Bessey a silver tea-service, bearing a suitable inscription, the nursing staff a marble clock, and there were other presents. It is pleasing to learn from the recipient of these gifts that she has spent an extremely happy time in the hospital, and it is very significant that she attributes her happiness to four things—the intense interest she had felt in the work; the gratitude of the patients, many of whom had been wont after their recovery to come in and have a word with her; the willing co-operation of the nurses who had served under her; and the consideration which she had always received from those who were in authority. We think that Sister Bessey has proved herself a nurse to the manner born.

### PRINCESS CHRISTIAN'S TRAINED NURSES.

In the nineteenth annual report of Princess Christian's Trained Nurses, which has just been issued, it is stated that during last year 711 cases were nursed in Windsor, Eton, and Clewer, and 15,739 visits paid, including attendance on 220 midwifery cases. Mention is made of the fact that, in consequence of the increase of the district midwifery practice, a small Maternity Home has been opened in connection with the Institute, where poorer patients are admitted on very moderate terms; and the report quotes the testimony of the inspector from the Jubilee Institute for Nurses that the result of her inspection was very satisfactory.

### STALLHOLDERS AT THE ELIZABETHAN FAIR.

Among the stallholders at the Elizabethan Fair and Fête on May 23, 24, and 25, in aid of King's College Hospital, will be the sister-matron, sisters, and nurses of the hospital, who with Mrs. A. C. Headlam and other ladies will be responsible for "The Old Cross at Enfield" in the grounds of Lincoln's Inn. This stall will be devoted to flowers and farm produce. At another stall of plain work and miscellaneous articles the Countess Roberts and her daughters will be assisted by the matron and nursing staff of the Queen Alexandra Military Hospital at Millbank.

### BRITISH NURSES' ASSOCIATION.

Questions of interest will arise at the annual meeting of the Royal British Nurses' Association on May 31. The meeting will be held at 3 p.m. in the Australasian Room of the Imperial Institute, and a reunion of members will take place afterwards at the Austrian Exhibition, Earl's Court. The resignation of five of the Lady Consuls—Miss Forrest (of Bournemouth), Miss Hunter (of Walthamstow), Miss Laurence (of Salisbury), Miss Cappe (of Chichester), and Miss Wills (of Newark) —is mentioned in the report as due to their inability to advise other nurses to join the Association, owing to the fact that they do not consider the Registration Bill approved by the Association likely to further the best interests of the nursing profession.

### BIRMINGHAM GENERAL HOSPITAL LEAGUE.

The first meeting of the League of Nurses at the General Hospital, Birmingham, has just taken place. A large number of past and present workers attended, and had the pleasure of meeting and renewing acquaintance with old friends. The object and rules of the League were explained by Miss Jones, the matron, in a speech welcoming her former sisters and nurses. A committee and sub-committee, etc., were chosen by election, and the League started on a businesslike basis. The subject of a badge was brought forward and a suitable one selected with the motto "Unity" attached. After the business was completed the social element prevailed and a delightful afternoon was spent in comparing notes as to the different experiences of members who had taken up work away from their old hospital.

### HOME FOR NURSES AT KESWICK.

It has been arranged to have a home this year at the Keswick Convention for a number of hospital nurses who have never been to Keswick. A chaplain, who is a medical man as well as a clergyman, will be in attendance, and his wife will act as hostess. She will be assisted by the Hon. Secretary of the Nurses' Prayer Union of the London Hospital. The Convention takes place in July.

### A FRIEND OF NURSES.

The most notable of many public bequests by the late Miss Claudia Griffiths, of Neath, amounting in all to £40,000, is the munificent sum of £5,000 to the Neath Nursing Institution. Miss Griffiths has also left £1,000 to the Royal National Pension Fund for Nurses; thus showing that while her interest naturally centred in the local nursing institution, it extended to a great movement for the benefit of nurses in general.

### CLAPHAM MATERNITY HOSPITAL.

The annual meeting of the Clapham Maternity Hospital was held on Friday afternoon last. The chair was taken by Lady Hamilton, who in her opening speech alluded to the admirable work done by Dr. Annie McCall, and suggested a "Rosemary Band" being started to try and get more help in the way of money and clothing. The annual report, which shows continued progress, was read by Miss Ritchie, Dr. Sturge was re-elected to the chair, and the whole of the Committee were re-appointed. Dr. Annie McCall spoke of the great need of better accommodation, the present hospital being quite inadequate. A site has already been obtained but funds are urgently required. Miss Le Geyt offered £5 on condition that twenty others would do likewise, and this was responded to by four ladies present. After tea an inspection of the hospital was made, including the new waiting home which has recently been opened.

### THE NURSES OF CAMBERWELL POOR-LAW INFIRMARY.

At the meeting of the Camberwell Guardians last week the Chairman personally congratulated the eighteen nurses who had successfully passed their examination at the Camberwell Infirmary. Mr. F. C. Abbott, in his report, stated that he examined twenty-two candidates, only four failing to pass. He found the nurses very well taught and thoroughly up to their work, and their teaching reflected great credit on the medical superintendent and his staff. Each of the successful candidates secured 45 per cent. of the marks on all subjects, their names being Nurses Carver, Burgess, Stavert, Black, Chapman, Mackenzie, Bath, Humphrey, Gwinnette, F. Heap, Stewart, Tyrer, Wilson, Sanders, Hall, M. C. Heap, Backhouse, and White. The medal, given by the medical superintendent, was awarded to Miss Burgess, and Miss Stavert passed first in the examinations.

### WORKMEN AND QUEEN'S NURSES.

At the opening of a bazaar in aid of the funds of the Sunderland Nursing Association, Mr. Alderman Bruce, who presided, stated that the work of the Queen's nurses in Sunderland had been so highly appreciated that at the present time there are no fewer than thirty large yards and works in the town where the men have voluntarily agreed to contribute a weekly, or a monthly, sum towards the upkeep of the institution. The bazaar itself was held in order to help to provide means for the purchase of another house to increase the accommodation necessary for the nurses. In places where such a spirit as that indicated by the action of the industrial classes prevails, and there is also an energetic committee, there is not likely to be much difficulty in respect to funds.

### A NURSING INSTITUTE FOR TIVERTON.

A useful gift has been made to Tiverton by Mr. John Coles. He has presented premises to the Infirmary, which are to be utilised as a nursing institute, and has supplemented it by a donation of £500, to be used for carrying out the needful alterations and providing the fittings. When this work is carried out it is proposed to enlarge the children's ward and improve the mortuary. A sum of £300 will be required for the purpose and for the furnishing of the new nursing institute, to raise which a fancy fair will take place next year.

### SHORT ITEMS.

The annual general meeting of the University College Hospital Ladies' Association will be held at the hospital on Wednesday, May 23, at 4.30 p.m., the Marchioness of Salisbury in the chair.

### The Nursing Outlook.

"From magnanimity, all fears above;
From nobler recompense, above applause,
Which owes to man's short outlook all its charm."

### FAULTY COMMITTEES.

The English system, or want of system, whereby the management of a hospital is vested in the hands of a committee selected from the governors, works satisfactorily from the standpoint of efficiency when the chairman is a man of affairs, and a few of the members have acquired an intelligent knowledge of the business and working of the institution. When, however, a committee is too large, or its chairman is too old, or lacks administrative gifts, the results frequently leave much to desire. On the whole, we believe it to be true that the matron and lady superintendent, of all officers, are liable to suffer most from a faulty committee. Formerly in London it was very difficult to secure an adequate number of capable laymen to serve on committees. In recent years, thanks largely to the example of the King and the Prince of Wales, the efficiency of the committees of Metropolitan hospitals has very greatly increased. In the larger provincial and Scotch cities the same holds good, but in the county towns and smaller communities faulty committees are not infrequently to be found still.

Let us take a concrete case which has recently led to inefficiency. To understand it it may be well to state that the practice still holds in some county hospitals to regard the house surgeon as the master of the house, and to put the matron and all the other resident officers under his control. This plan is a remnant of days when the house surgeon used to be a man of some standing in the profession, who remained for several years in charge of a county hospital with the object of settling in the town as a surgeon or physician, when he secured election as honorary medical officer. In such circumstances the system worked smoothly on the whole. But times have changed, and it now not infrequently happens that the house surgeon may be recently qualified, without experience or the wider discretion which comes with years. For this reason it does not make for efficiency to place the matron and her staff under the house surgeon, especially when there is a training school for nurses attached to the county hospital. In such an establishment it is essential that the matron should have control over the whole of the female staff, for otherwise she cannot enforce discipline or maintain her position, especially under a weak or faulty committee.

We have known a case where a matron on her night rounds found the resident medical officers smoking in the day-room with the night nurses in attendance, and, when her orders and remonstrances were resented by the resident medical officers the committee, through weakness, failed to appreciate the situation, and so cast the weight of their authority against the matron, who merely did her duty. It is essential, too, that the medical attendance on the nursing staff should be entrusted to a senior member of the profession, and not left in the hands of the junior resident officers. No matron who was really efficient would undertake the superintendence of a county or other hospital of any size unless the regulations gave her adequate control over the whole of the female staff. It is, however, very hard on a competent lady superintendent, who may have worked zealously for years in an institution, to have her discipline upset and her authority flouted by some recently qualified junior, who may have been appointed house surgeon. Of course when a committee is efficient difficulties of this kind seldom or never occur; but a weak chairman or a faulty committee may keep the internal administration of a hospital in a state of chronic inefficiency for years and perpetrate petty injustices to its resident staff from mere want of energy and knowledge.

We have called attention to these matters owing to some recent instances which have been brought to our notice where injustice to officers has been perpetrated, with the result that inefficiency must continue to prevail as the direct consequence of the ineptitude of the committees. We hope that those matrons who may be suffering from causes such as those we have indicated will take an opportunity of moving their friends on the committee to bring the matter forward, and get the regulations modified so as to vest them with control over the nurses and female staff. If it be held essential that the senior resident medical officer shall be master of the household, then it is the duty of the committee to offer such an officer a salary of sufficient amount to command the services of a man of experience with administrative gifts. The trouble is usually caused by the careless selection for these posts of a student without experience, who has only recently qualified. It should be easy to avoid mistakes of this kind, and it will be well for the chairman of committee of many a county and smaller general hospital to confer with his matron and, as a matter of business, go into the regulations of the various officers with a view to bring them on to modern lines and to make them so clear as to vest an adequate amount of responsibility in the hands of each of the responsible officers so as to secure the maximum of efficiency not only in each department, but throughout the whole institution.

The Committee of any county or other hospital who fail to appreciate an administrative fact of this importance are sure to act unjustly to the matron and cannot hope to make the hospital popular with the public and patients. They may act in good faith no doubt but the harm their want of judgment may cause is often incalculable and mischievous.

### The Care and Nursing of the Insane.

By Percy J. Baily, M.B., C.M.Edin., Medical Superintendent of Hanwell Asylum.

### I.—ANATOMY AND PHYSIOLOGY.

(*Continued from page 77.*)

*The Classification of Foods.*—The subject of the chemical composition of the tissues is a very complex and difficult one, with which we need not concern ourselves here. It is necessary to explain, however, that there are certain chemical elements in our tissues which must be supplied to them in the food we eat. Of these, four which enter into the composition of organic foods (foods, that is to say, which are derived either from animals or plants) are all important. They are oxygen, hydrogen, carbon, and nitrogen. In order that life may be sustained these four elements must exist in the food we eat in certain definite proportion. In addition to these we require certain minerals which exist in the food in the form of salts.

However varied may be the articles of food we take, they may all be grouped according to their chemical composition under four heads, which are:

1. *Nitrogenous or Albuminous Food-stuffs.*—These foods are compounds which contain all the four elements mentioned above—namely, oxygen, hydrogen, carbon, and nitrogen. As examples of this class of foods may be mentioned all kinds of lean flesh, such as mutton, beef, veal, chicken, fish, etc., and also the white of eggs and cheese.

2. *Fats.*—These contain no nitrogen, but are compounds of the other three elements, oxygen, hydrogen, and carbon. The fat portion of meat, suet, lard, butter, and the yolk of eggs, are examples of this class of foods.

3. *Starches* and *Sugars.*—These, like the fats, contain only oxygen, hydrogen, and carbon, but in different relative proportions. They form a most important class of foods. Wheat flour (bread), cornflour, rice, potatoes, cane sugar, etc., are examples.

4. *Various Mineral Salts* and *Water.*—Water enters so largely into the composition of the body (it constitutes about two-thirds of the entire weight) forming the bulk of the blood and lymph that we require to take very considerable quantities of it. Much of the solid food we eat contains large quantities of water, especially some vegetables, such as turnips and fruits. In the body, water acts as a solvent, and carrier, and by its agency, many waste products are removed as in the urine and sweat. The mineral salts include common table salt (chloride of sodium), salts of potassium, calcium (lime), and iron, and many others.

Let us now make some inquiries into the nature and arrangement of the organs we possess for dealing with these food-stuffs, and learn what we can of the manner whereby the changes in them necessary to render them soluble are brought about.

*The Anatomy of the Digestive System.*—This must include a brief description of the alimentary canal, and also of those glands, the salivary glands, the liver, and the pancreas, which, although they lie outside the digestive tract, pour their secretions into it.

*The Alimentary Canal* is a tube, in all about 30 feet long, which passes through the body (fig. 15). It commences at the mouth and terminates at the anus. Its walls are composed of three distinct coats

| Classes of Foods. | Digestive Juices. |
|---|---|
| 1. Nitrogenous (meat, &c.). | 1. Saliva. |
| 2. Starches and Sugar. | 2. Gastric Juice. |
| 3. Fats. | 3. Bile. |
| 4. Salts. | 4. Pancreatic Juice. |
| 5. Water. | 5. Intestinal Juice. |

Fig. 15.—Diagram of the Digestive Apparatus.

1. Mouth: Food masticated and mixed with secretion of salivary glands (saliva) which changes starches into sugar. 2. Pharynx: Walls contain voluntary muscle. 3. Œsophagus: Walls of this and all remaining parts of alimentary canal contain involuntary muscle. 4. Stomach: The mucous membrane contains the peptic glands which secrete pepsin. This dissolves nitrogenous foods, converting them into peptones. 5. Duodenum: This part of the small intestine receives the secretions of the liver (bile) and pancreas (pancreatic juice). The pancreatic juice changes starches into sugar and nitrogenous foods into peptones, and it emulsifies fats. The bile assists the pancreatic juice in emulsifying fats. It also facilitates the absorption of fats into the lymphatics of the intestine. 6. Small Intestine: A tube about 21 feet long. Its mucous membrane contains glands which secrete the intestinal juice. This continues to carry on the process of digestion, and probably acts like the pancreatic juice but more feebly. 7. Appendix. 8. Large Intestine or Colon: No further digestion takes place after the food leaves the small intestine, although absorption still continues during its passage through the colon. 9. Rectum.

N.B.—The products which result from the digestion of starches (sugar) and nitrogenous foods (peptones), also the water we drink and the soluble salts of the food, are absorbed directly into the blood vessels (capillaries) of the stomach and intestine, but the fats enter the intestinal lymphatics.

or layers. Throughout its whole extent the inner surface of the tube is lined with mucous membrane. Outside this is the muscular coat, composed of layers of involuntary muscle fibres, and covering this coat, in the abdominal portion of the alimentary canal, is a very thin, delicate, glistening membrane called the peritoneum. It is this membrane which becomes inflamed in the disease known as peritonitis. The peritoneum also lines the inner surface of the walls of the abdomen.

*Mucous Membrane* is found in other parts of the body besides the alimentary canal--for instance, in the air passages, as we shall see when speaking of the respiratory system, and in the bladder, etc. At the lips the general characters of mucous membrane may be compared to those of the skin. It forms a much softer and more delicate covering than the skin, and its surface is kept continually moist by the secretion of a slightly viscid fluid which is poured out from innumerable minute glands (the mucous glands) which are embedded within it. In addition to these glands it contains a very large number of blood-vessels, its red colour being due to this fact. In the stomach and intestines the mucous membrane contains, in addition to the ordinary mucous glands, many special glands which secrete the digestive juices.

*The Mouth* is the cavity into which the food is received. It is when closed almost, if not entirely filled up by the tongue, which lies upon the floor of the cavity. The tongue is a mass of muscular tissue which is covered by mucous membrane. Within this mucous membrane there end the special nerves of taste. But besides being the organ of taste, the tongue has other functions. It is very sensitive, so that so slight an object as a hair can easily be detected by it; and hence, if any hard or sharp object enter the mouth with the food, such as a small fish bone, for instance, the tongue gives warning of the fact, and so enables us to remove it from the mouth. In the first stage of the act of swallowing, the tongue, as we shall presently see, plays a very important part; and lastly, it is by means of the movements of the tongue (and lips) that we pronounce many of our letters; hence we find, when the tongue and lips become paralysed, as they do in general paralysis, for instance, that the patients have great difficulty in pronouncing many of their words and in starting the act of swallowing.

The entrance of the mouth is guarded by the lips, while its sides are formed by the cheeks. Its roof constitutes the palate. The front part of this is called the hard palate, for it is composed of bone covered by periosteum and mucous membrane. But the hinder part of the palate consists of muscle, covered by mucous membrane, and this part is known as the soft palate. The central portion of the soft palate is prolonged into a pendulous tassel called the uvula.

At the back the mouth is continuous with the pharynx or throat, and on each side of the passage between the two is a gland called the tonsil.

Immediately within the lips and cheeks are the teeth, arranged in two semi-circles, an upper and a lower semi-circle. They are fixed into the bony edges of the upper and lower jaws, which are covered by the gums, which consist of periosteum and mucous membrane. The teeth have different shapes, according to their uses. Those in front, both in the upper and lower jaw, have sharp, straight edges, so that when they are brought together, as in the closing the jaws, they cut through anything which may be lying between them, after the manner of a pair of scissors, hence they are called incisor teeth. There are four incisors in each jaw, and next to them comes on either side a pointed tusk-like tooth called the eye tooth or canine. Behind the canines are the premolars (two) and the molars (three). These latter ones have more or less flat crowns, and their function is to crush the food after the manner of millstones during the process of mastication. In the first set of teeth—the milk teeth, as they are called—there are only two behind the canines on each side in each jaw, so that in young children there are in all 20 teeth, whereas adults have in all 32.

The secretion of the salivary glands is conveyed into the mouth by means of ducts. Some of these glands lie in the floor of the mouth between the tongue and the lower jaw. Others lie just in front of and below the lobe of the ear (parotid glands). These latter glands often swell up to an enormous size in the disease called mumps.

### The Nurses' Clinic.

### VESICO-VAGINAL FISTULA CASES.

A vesico-vaginal fistula is generally the result of some injury to the bladder. It may be caused during an instrumental labour, or a very prolonged labour, by pressure of the child's head, after which sloughing has taken place. Sometimes it is due to malignant disease. Patients who suffer from this complaint are subject to great inconvenience. The urine dribbles away all the time, the bladder having lost power of retention, either partially or completely.

Occasionally the surgeon may succeed in closing the fistula, if small, by cauterisation, but if it be large he performs a plastic operation. The preparation of the patient is the same as for most other vaginal operations. An aperient is given about midday the day before, followed by a soap and water enema and vaginal douche that evening and early the next morning, a wash out enema and vaginal douche being given within an hour before operation. The parts must be shaved and the patient must have a bath. Breakfast should be very light and should be given about four hours previous to operation. The patient must be catheterised immediately before going to the operating theatre, so as to make quite sure of the bladder being empty. She will wear a warm dressing-gown and stockings, and will be operated on in the lithotomy position, the legs being supported by crutches.

The necessary instruments, etc., will be: Scalpel, long uterine scissors, probe, duckbill speculum, small curved needles, needle holding forceps, two pairs of dissecting forceps, half a dozen pairs of pressure forceps, sponge holders, swabs of sterilised gauze, urethral dilators, glass catheter, Bozeman's catheter, douche nozzle and irrigator filled with antiseptic solution, catgut and silkworm gut sutures, large plugs of iodoform gauze, pads of gamgee and T bandage.

The after-nursing of these cases varies according to the surgeon. Some surgeons have the patient, after she has recovered consciousness from the anæsthetic, placed face

**THE NURSES' CLINIC**—*Continued.*

downwards in bed; this position is a very trying one to lie in, and a great deal of care must be taken by the nurse to protect the knees and arms from pressure, pillows and rings of wool being placed under the patient so as to make her as comfortable as possible under the circumstances. Sometimes a catheter is passed into the bladder and left there, being fixed in position with strapping plaster attached to the patient's legs. The nurse must fix a piece of tubing to the end of the catheter and place a small vessel underneath to catch the urine. In this way the bladder is kept empty all the time so as to save any strain whatever on the part where the fistula has been.

The patient is turned over on her back about three times in the twenty-four hours, when the catheter is taken out, cleaned and boiled, and then replaced, a vaginal douche being given of warm boracic solution. She lies in the face downward position for about ten days; if the stitches are of silkworm gut they are then removed by the surgeon, if catgut they will have absorbed, and the patient is allowed to get up.

Some surgeons dilate the urethra at the time of operation and allow the patient to pass urine herself from the first, others have her catheterised every four hours. In some cases the surgeon packs the vagina with iodoform gauze, which is removed by the nurse as ordered, the day or perhaps two days after operation and vaginal douching commenced. An aperient is usually given as in any ordinary case the day after operation, and after that when necessary. The patient may have light ordinary diet. These cases are often difficult to heal and may have to be operated on several times, which is disappointing to the patient as well as to the surgeon and nurses.

### The Best Method of Keeping Helpless Patients Dry.

### EXAMINATION QUESTIONS FOR NURSES.

The question was as follows: What measures would you take to alleviate discomfort and avoid evil consequences in the case of a bed-ridden patient, male or female, who suffers from incontinence of urine. N.B.—You are to consider the house to be a poor one and appliances and comforts few.

### The First Prize.

In cases of incontinence of urine one mackintosh at least must be procured. It is better to have two, but in a poor household this is not always possible, so one must manage with one. This must be covered by a fairly wide draw-sheet. If possible, a water-pillow should be procured.

It is better not to put pads of wool or tow under the patient, as these encourage a tendency to bed-sores. The draw-sheet must be changed as often as necessary, and each time this is done the mackintosh should be wiped over with a solution of carbolic 1 in 20. This will keep it fresh and clean, and do away with that obnoxious smell that nearly always accompanies cases of incontinence.

The patient's back and buttocks should be thoroughly washed each time with warm water and soap, and then dried; some zinc and benzoin ointment should then be well rubbed in with a brisk circular movement.

If the washing process is done thoroughly each time the sheet is changed the tendency to bed-sores, which is the greatest danger in these cases, will be greatly minimised.

Special care should be taken to keep the folds of skin in the groin dry and well powdered with zinc oxide and starch powder.

It will greatly add to the comfort of the patient if instead of the ordinary nightshirt or nightdress a short jacket were worn, as this will not get wet, and also will greatly economise the washing.

Keff.

### The Second Prize.

In a case of incontinence of urine in a bedridden patient I should see that the mattress was protected from urine by some waterproof material, if procurable; if it were not, the use of some thick brown paper, enveloped by muslin (this last may be had for the asking almost at a provision or butter dealer's, and will answer the purpose—namely, to keep the paper from sticking to the bedclothes when wet) will keep the wet from the mattress if looked after regularly and changed as soon as damp.

Any old sheets with the holes or rough parts turned in so as to get a smooth surface, I should use as draw-sheets, these could be washed and dried ready for use again as soon as possible.

For a male patient I should get a child's tin pail and fill with sawdust and place between the legs so as to catch the urine as passed, this would not be possible in all positions, but could at all events be used while the patient was lying on his back; at other times I should get some old hand-towels, or some other soft absorbent material, and place in such a position as to soak up as much as possible of the urine without its coming in contact with the patient.

I should have in lieu of a urine bottle a three-pound glass jam jar and encourage the patient to use this at intervals, unless the urine was dribbling away all the time, when it would not be of much use.

To prevent bed-sores I should see that the bed was kept dry, the sheet smooth and free from crumbs, etc.

I should sponge the patient all over twice a day if possible, and those parts likely to be affected by the urine more often, dry carefully, and any parts looking red, and those parts mostly in contact with the bed I should gently massage each time the patient was washed. The parts most likely to come in contact with the urine, scrotum, buttocks, etc., I should smear with a little zinc ointment; I should encourage the patient to change position as often as possible, or, if unable to do so himself, help him to move from side to side and back at intervals, not allowing him to lie too long in one position.

Nurse G.

### The Prize Winners.

"Keff" is successful in winning the first prize because, besides mentioning various things that can be done to ease the sufferer, she warns nurses against the plausible sounding but really dangerous practice of putting pads of any kind under the patient to soak up moisture. She also speaks of the necessity of great care in washing and tending the groins, etc. Constant care of this kind must always be given by the nurse if she would avoid an inflammatory state of the skin appearing after a week or two. She mentions no manner of utilising any kind of urinal—it is true that if it is possible to use such an article, the necessity for so many precautions vanishes; still it would be more satisfactory to find that her contriving genius could if necessary be brought into action.

The winner of the second prize, "Nurse G.," is full of resources, and the muslin over the brown paper is an excellent idea, as constant wetting even for a few hours causes the surface of brown paper to adhere tenaciously to linen.

### Honourable Mention.

This is gained by "Clarence Belfast," "Busy Bee," "Nurse," and "Tweenie." It would be well if candidates would think out their pseudonyms a little. "Nurse" may be anyone, as all competitors are nurses!

The chief fault in all the papers is that the subject has been treated too much as if it were one concerning the prevention of bed-sores; this is a mistake—of course, with such a condition of things there is that danger, but it is equally important in all cases to maintain a healthy state of skin around the external genitals, groins, and thighs. Where the urine is very irritating it is well to protect these parts by pieces of lint or linen well spread with ointment. This will not alter the necessity for frequent washing, but it will greatly protect the skin.

Question for May.

What arrangements—in a poor cottage—should you make in the case of a heavy patient suffering from heart disease and dropsy to aid in the constant moving so pitifully desired by those suffering from these maladies.

The Examiner.

### Incidents in a Nurse's Life.

### A CASE OF HYDROPHOBIA.

He was a new patient, a big strong young blacksmith in the prime of life, and I was his "special" for the time being. As I sat beside his bed, finding that it relieved the poor fellow to talk, I allowed him to recapitulate the story of his illness from the beginning, and tried by my silence and my expression to show him how I sympathised with him.

"Six weeks ago," he said, "I was playing with my Persian cat, and a great pet she was; such a beauty too! and what made her do it God alone knows, but she snapped at and bit my two middle fingers. It was not much to look at—little more than severe scratches—but it hurt me, so I went to a chemist and he cauterised the fingers. I had made such a pet of my handsome cat that it went sorely against the grain, but some days later mother and I both thought it would be safer to kill her, as she seemed so spiteful and bad tempered. My fingers healed. I felt no pain, and had begun to forget the affair, save for the marks left upon them.

"Then one evening I felt a peculiar shiver and horror come over me, and at supper, as I raised the cup to my lips or the bread to my mouth, I had the utmost difficulty in making myself take the sip or the bite. You will ask me why? I cannot describe to you my terror of mind. Of what? You may well ask. You know how undefinable a dream is—how you may go through horrors that are difficult, nay impossible, to describe. So it is with me now.

"This awful terror seized me two and a half days ago, and as I got worse my mother and the doctor urged me to come here to the hospital. I now know the name of my disease, and feel that each hour I get worse. These spasms of shivering, shuddering terror come oftener and stronger, although my mind is so clear. Can nothing be done for me? Look at me, nurse, in perfect health; look at the muscles of my arms, look at my size, my strength, my youth. Yet here I am doomed to this awful death. It is not fit for you ladies to be with me: Go, go, I beg you."

As the patient had been speaking he grasped my arm as in a vice of iron. I showed no fear, but wondered what might come next. So when he said "Go," without further argument I went and reported his wishes. Then it was arranged for two men who had acted as orderlies in the Army to watch by him, turn about. There was nothing "unfit," as he deemed it, for nursing sisters to see or hear, but the mental strain to us was great of this soul in its agony, and as the spasms increased in intensity he would start from his bed, and, clinging to the sides, plead and beseech someone to help him.

The room had been darkened and carpeted, as even a flash of light or a footfall on the floor would bring on a spasm. The visiting surgeon asked for some bread and milk to be made for him. I brought this in and lifted some to his mouth; but before the spoon actually touched his lips another terrible attack came on. Later he was asked if he would smoke; he was quite willing to try, and a pipe was lighted. This he also took in his hand and brought it near his mouth—but not near enough to touch the lips—when the same thing happened again. This awful mental terror and spasm! A mental and physical rigidity making it impossible for him to either eat or drink. In piteous accents of despair he sought the surgeon's help. We soothed him all we could. Alas! none could help him. And as his condition became more violent (he would expectorate anywhere), it became necessary to lesson his sufferings with an anæsthetic, until he finally drew his last breath just twelve hours after his admission into the hospital, and on the third day from the symptoms first declaring themselves, which is, I believe, the usual, if not the invariable, time the illness lasts. Words quite fail to express this distressing and happily rare complaint. Though doctors and students would have given much to see the "case," none were admitted to the room beyond those actually in attendance on the patient. The extreme clearness of intellect was a marked and painful feature. Had the exquisite torture the poor fellow suffered been witnessed by those who are keen anti-muzzlers, I think their conversion would have been speedy and lasting. Even in far away Northern Rhodesia I have often found the dogs muzzled.

### Sir William Bennett on Nursing Associations.

Princess Louise, Duchess of Argyll, was present at a meeting at the Mansion House on Wednesday last week in support of the South London District Nursing Association. There was a good attendance, and the chair was taken by the Lord Mayor, who subsequently explained that having some years ago acted as hon. auditor to the Association, he had for that reason agreed to a meeting being held in the Mansion House in its support.

Canon Erskine Clarke, who was the next speaker, remarked that the nurses of this Association looked after people who were either not sufficiently ill to go into a hospital, or had recently been discharged from one. The visit of one of these nurses was looked upon by the poor people as the visit of a ministering angel. The Association was deserving of all the support it could get.

Mr. J. F. Schwann, Hon. Treasurer, stated that the work had no paid officers and no expenses of any kind; all the money went to the salaries—he might say the pittances—paid to the nurses, and towards the household expenses. These last were not extravagant, the cost per day coming out at 9d. or 10d. per head.

Sir Joseph Dimsdale said that the Association had been doing good but unostentatious work for the last 22 years among the poor of that vast and teeming Metropolis. Great as the work was that it had done, an almost unlimited field lay before them, if only they had sufficient resources placed at their disposal. Many lives might be saved if only they had more nurses.

Sir William Bennett said that it was chronic disease which broke the back of the poor. Cases of that kind could not get into the hospitals. This was, in his opinion, one of the blots on modern hospital administration, and must remain so as long as the charitable public were led to understand that the success of a hospital depended upon the number of patients that passed through its doors, forgetting that numbers were no test at all of efficiency or of the actual amount of work done. Nursing associations kept the patients suffering from chronic disease out of the workhouse infirmaries. There should be a great combination of parochial authorities to bring about a widespread organisation of nursing associations.

Archdeacon Wilberforce also spoke of the extraordinary humanising influence of the nurses in the homes of the sick poor.

### East-End Mothers' Lying-in Home.

There was a very successful gathering of the friends of this Home at 3 Grosvenor Place on Friday. The chair was taken by Mr. Owen Lankester, who, in his opening remarks, said that the Home had increased in usefulness, and was even better managed than before. He invited his hearers to come and pay a visit to the institution and see for themselves the mothers in their ease and the babies in their comfort. During the past year various improvements had been effected; a lift had been added, the wards had been painted, and electric light installed. They were in need of funds for these additions. Finally, he said that their new Matron, Miss Margaret Anderson, had done splendid work in the past year.

Lord Oranmore and Browne moved the adoption of the report, and remarked that when they considered the contrast between the bright rooms and the comforts of the Home and the squalid dwellings from which these poor mothers came, it was no wonder that many of them said the only holiday of their life was the time they spent in the home. The Committee were anxious to raise enough money to purchase one of the two houses they occupied. They had now an excellent sink-room supplied by the generosity of Miss Dodge. They had only had one death out of the 382 cases received in the home during 1905, and 377 children had been born alive and only eight still-born. He then paid a warm tribute to the work of the staff, and said that the Committee owed a deep debt of gratitude to the Visiting Medical Officer, Dr. Corner, and also to their excellent Matron, whose aim it was to make the time passed by the patients in the home a really happy one.

The Hon. Mrs. Emmott Barlow seconded the motion, and described a visit she had recently paid to the home, when she saw the food being prepared for the patients, nicely arranged on little trays with napkins on them. The training given to midwives at the home must be considered very satisfactory, seeing that the home ranked second on the County Council list after Queen Charlotte's Hospital. Commenting on the ignorance of poor mothers, she instanced the fact that "cinder tea" was considered among them the best remedy for flatulence in infants.

The Hon. George J. Goschen moved a resolution stating that the home was doing excellent work, and deserved wider financial support. Observing that every year hygiene and science made greater strides, which always meant more expense, he said he trusted that the result of the meeting would be that they would obtain increased support. The Samaritan Fund, which supplied food and clothing to out-patients, stood sorely in need of money, and the Matron would be extremely grateful for further assistance. He was confident that if people would see for themselves the comfort and happiness the Home gave to the poor women they would assist the work with the utmost liberality.

Dr. H. R. Andrews seconded the resolution, which was carried.

### Presentations.

On May 4 an interesting presentation took place at the Trained Nurses' Institution, Hyde Terrace, Leeds, when the nurses presented the lady superintendent, Miss Dawson, with a pearl and diamond brooch, in commemoration of her thirty years' admirable work at the institution from the time it was first opened in 1876. They also marked their appreciation of the kind and attentive services of Dr. E. Roberts, honorary medical officer, for over seven years, by presenting him with a handsome silver inkstand.

### Everybody's Opinion.

[Correspondence on all subjects is invited, but we cannot in any way be responsible for the opinions expressed by our correspondents. No communication can be entertained if the name and address of the correspondent are not given as a guarantee of good faith, but not necessarily for publication. All correspondents should write on one side of the paper only.]

### NURSING UNDER CHRISTIAN SCIENTIST AUSPICES.

"An Institution Nurse" writes: I am very pleased to see how severely the Coroner has censured the Christian Scientists! It is only a few years ago since Mrs. Eddy herself was charged with "manslaughter" in connection with the death of Mr. Harold Frederick. Recently I was nursing in a house where the lady was visited professionally by a couple who were Christian Scientists, and Buddhists, as well as astrologists. They told the lady her horoscope and that she would go to Mentone for Christmas, her husband would die and she would marry again. As it turned out, she spent her Christmas at home and her husband is still living. By way of "medical" advice htis couple also sent their photographs to her; the lady was to gaze at the man's photograph from the time she woke up in the morning till the afternoon, and at the woman's photograph from then till evening! I do not know how many guineas were paid for that "advice," but I fancy a considerable sum, as the horoscope alone cost six guineas. We private nurses often do hear of sad credulity and of much nonsense which is both amusing and sad.

### OUTDOOR UNIFORM.

"C. O." writes: I cannot refrain from writing a few lines in answer to "M.D." on the wearing of uniform. Her letter certainly adds greatly to the amusing reading she refers to. Why should we give up our uniform for servants to wear it? It is "nurses'" uniform and we have the right to wear it. I think it is little use making so much fuss about nursemaids wearing it. We all know that private individuals are at liberty to dress their maids as they think fit, and we cannot help them copying our uniform. One can easily tell them; there is a "something" lacking in the imitation that is at once visible. I hope that if the time ever comes that "M.D." wishes for, when nurses are debarred from wearing outdoor uniform, that my nursing days will have ended. There are other nurses I am sure, besides myself, who will not care about giving up their uniform so that servants may wear it. Imitation is the most sincere form of flattery.

### IRISH NURSES AND REGISTRATION; THE THREE YEARS' CERTIFICATE.

A "Dublin Hospital Nurse" writes: I am anxious to get some information on the following questions which are of great importance to all nurses trained in Ireland, and upon which most of us are at present totally in the dark. I understand that the authorities for State Registration will require nurses to have certificates for three years' hospital training before they can be registered. Does that mean that they must have three years' consecutive training in one hospital, or, if a nurse has a certificate for two years' training, and in addition can get another year's training in the same hospital (and a certificate to that effect), or failing that an extra year in any other recognised training school, will she then be qualified for State Registration? Most hospitals in Ireland train their nurses for only two years in the wards, and no matter how many years we may afterwards serve our respective hospitals, either in hospital or on the private nursing staff, we get certificates for only "two years' training" at the end of our terms of service; consequently, when we apply for appointments afterwards at other institutions—either in Ireland or elsewhere—we find ourselves disqualified by reason of our certificates. Take, for example, the "Services," each of which requires candidates to have certificates for "three years' consecutive training" in the wards. Again, 99 per cent. of the advertisements of posts for trained nurses insist that the candidates "must have had three years' training." Will the superintendents or committees who advertise these posts (I presume that they are the criteria in this case) accept as eligible for the appointments, nurses who have obtained their three years' training at intervals, or do they consider the "consecutive" training indispensable? What then is to become of nurses who have only "two years" certificates? We find at the end of our terms of service that we (in most cases) have not only spent on our premiums all the money we had, but also at least four of the best years of our lives, to obtain certificates which, for all practical purposes, are not worth the paper they are written on. Shall we be disqualified for State Registration also? These are questions which we Irish nurses are most anxious should be clearly understood and answered once for all. We feel that we are labouring under great disadvantages, and we cannot understand why we should be deprived of what we justly consider as our right, *i.e.* to have certificates which will place us on an equality with the nurses of England and Scotland, which at present we certainly are not. There is much more to be said on this subject, but I hope that this is enough to interest in our cause those who have influence in bringing the standard of nurses in Ireland up to what it should be.

### PRACTICAL HINTS.

"M. C. W." writes: It would very much add to the interest and value of the articles entitled "The Nurses' Clinic" and "Practical Hints," if the writers, when mentioning any fresh simple treatment, would state the quantities of the ingredients. For instance, in the article on "Eczema" the writer says how efficacious a starch poultice is. Readers would have been glad had the exact amount of starch and water used been given, if necessary, in a footnote. I may add here a very simple treatment for abdominal sickness. The patient was a Hungarian, and whilst in Vienna he had severe sickness from appendicitis, so that his doctor said he must go into the hospital and sent a nurse the previous night to prepare him. She at once gave him tiny pieces of ice to suck, and then told him to take very deep breaths. This at once stopped the sickness. In the morning, when the doctor came, he said that the crisis had passed and that the patient need not be taken to the hospital at all. Since this is such a very simple remedy, I think it might be worth while for some nurse to try it. I have had the pleasure of nursing much among foreigners, and it is most interesting learning about their different ways and methods.

### Appointments.

Chesterfield Workhouse Hospital.—Miss Mary Sissons has been appointed night sister. She was trained at Toxteth Park Workhouse Infirmary, West Derby.

Ellon District Epidemic Hospital.—Miss Minnie Wilson has been appointed matron. She was trained at the Royal Infirmary, Aberdeen, where she has since been staff nurse and temporary ward sister. She has also been attached to the staff of the Glasgow and West of Scotland Co-operative Society of Nurses.

Gilsland Convalescent Home.—Miss Emma Thornton has been appointed matron, and Miss C. Naylor assistant matron. Both were trained at Bradford Infirmary, and hold the Central Midwives certificate. Miss Thornton has been assistant matron at the Convalescent Home, Gilsland, for four years, and Miss Naylor has been ward sister and assistant superintendent nurse at Bagthorpe Infirmary, Nottingham.

Infectious Diseases Hospital, Burslem.—Miss Edith Dawson has been appointed charge nurse. She was trained at the London Fever Hospital, and has since been staff nurse at the Isolation Hospital, Warrington. She has also done temporary work at other hospitals.

Johnson Hospital, Spalding.—Miss Eleanor Dawson has been appointed staff nurse. She was trained at the Walsall and District Hospital, and has since been staff nurse at the Accident Hospital, Ebbw Vale.

Keighley Isolation Hospital.—Miss Annie Hill has been appointed sister. She was trained at Bethnal Green Infirmary, and has since been nurse at Huddersfield Sanatorium and Tilbury Accident Hospital.

Lincoln County Hospital.—Miss Edith A. Wynne has been appointed matron. She was trained at Leeds General Infirmary, and has since been sister of the men's surgical ward, night superintendent, and assistant superintendent of nurses in the same institution.

National Institution and Molyneux Asylum for the Female Blind of Ireland.—Miss J. Reynolds has been appointed matron. She was trained at the Adelaide Hospital, Dublin.

St. George's Workhouse, Buckingham Palace Road, London.—Miss Lillian E. Allen has been appointed nurse midwife. She was trained at the West Ham Infirmary, Leytonstone, where she was afterwards staff nurse and ward sister. She was subsequently charge nurse at Joyce Green and Long Reach Small-pox Hospitals, Dartford, Kent, and charge nurse at the Eastern Fever Hospital, Homerton. She holds the certificate of the Central Midwives Board.

St. Pancras Infirmary (South).—Miss Nellie M. Rance has been appointed night superintendent. She was trained at St. Marylebone Infirmary, and has since been sister at St. Pancras Infirmary. She is a member of the Army Nursing Service Reserve.

The Bignold Cottage Hospital, Wick.—Miss Jean Sinclair has been appointed matron. She was trained at the Western Infirmary, Glasgow, where she has since been sister.

The Lady Forester Hospital, Much Wenlock, and District Nursing.—Miss L. A. Baskett has been appointed lady superintendent. She was trained at King's College Hospital, London, where she afterwards had charge of the ophthalmic wards. She has since been night superintendent at the Royal Hospital for Diseases of the Chest, City Road, London; home sister at the Bristol Royal Infirmary; sister at the Lady Forester Hospital), Much Wenlock; and matron of the Central London Ophthalmic Hospital, Gray's Inn Road, London.

Throat Hospital, Golden Square, London.—Miss Florence Bean has been appointed night sister. She was trained at the Seamen's Hospital, Greenwich, and the Hospital for Women, Soho Square, London.

Union Infirmary, Bridgwater.—Miss Alice M. Barnes has been appointed Superintendent nurse. She was trained at the Union Infirmary, Birkenhead, where she was afterwards charge nurse. She has since been sister at Leith General Hospital. She holds the certificate of the Central Midwives Board.

### Death in our Ranks.

An impressive funeral service was held last month in St. John's Cemetery, Wynberg, on the occasion of the interment, with full military honours, of Miss Margaret Kendall, a young sister of Queen Alexandra's Imperial Military Nursing Service who had been stationed with a branch of that service at the Military Hospital, Wynberg Camp. Miss Kendall was trained at Addenbrooke's Hospital, Cambridge, and she remained there until she joined the Imperial Military Nursing Service as one of twelve staff nurses chosen for the Royal Herbert Hospital, Woolwich. She served at Woolwich as staff nurse for eighteen months, and then as sister until she was ordered to South Africa in October 1905. She was only at Wynberg, Cape Colony, for six months, when she was taken suddenly ill while on night duty on April 2. She died on April 7 after undergoing a serious operation, and was buried on the following day, her twenty-ninth birthday.

### A Book and its Story.

### MRS. LITTLE'S NEW NOVEL.*

Mrs. Archibald Little, who knows China well, has chosen that country as the background to the love romance which springs from the chance meeting of two British subjects at Macao. This meeting between a girl of fifteen, Betty Formby, the only child of a consul-general at Hankow, and Trevor Laurence, a millionaire travelling in search of the picturesque, leads to the development of a very charming and unusual story. Betty is the companion of her clever father and having lost her mother when a child she has grown up content with his society. Being a girl of extraordinary quick perception, she realises among other things that her abilities are not recognised as they should be by those in authority at home. She expresses herself at all times with unrestrained frankness—which is one of her attractions—to Trevor Laurence. Speaking to him of her father she says ". . . . and they have not the least idea . . . what a great man he is under that little pleasantness—what a burning soul, what clearness of vision, what a grasp of detail, what a burning desire to help—all that makes a leader among men." Trevor Laurence is not a millionaire of the usual type. His money is not the result of his own exertions, but was left to him by his father, the son of a self-made man. Mentally gifted, he had taken a first class at Oxford; but, not being over physically robust, a tour through the capitals of Europe and a short Parliamentary career had, up to the time that the book begins, left him with the desire of further travel, to escape from some of the cares which his great wealth entailed. China appealed to him for many reasons. His very practical sister, Lady Morten, who had neither inclination nor capacity to share her husband's diplomatic position in St. Petersburg, makes a virtue of the occasion by declaring that "Trevor is not strong and needs someone to look after him," and decides to accompany him. She leaves her two boys, notorious pickles, at school. Their holidays are to be spent with a beautiful but impossible aunt, Lady Lillian Edgcumbe, their mother consoling herself by the reflection that their wild pranks might possibly keep their aunt out of mischief, and that the arrangements made for them would be sure to turn out well, those arrangements which left her free to start with her millionaire brother, to whom, in her loyalty and almost slavish devotion, "there was indeed something of the ancient Saxon serf's unquestioning devotion to his Norman chief." She dismisses them from her thoughts, after carefully packing their last photographs in her travelling bag, with one of Lord Morten, taken for a diplomatic series. "Lady Morten trusted the boys would not blow up the castle nor burn their own fingers too badly." The points of view between Trevor Laurence and his sister Lady Morten regarding the goal of their journey vary. "I don't quite know why you go to China," she said to her brother. "It seems to me a country there is no practical use in studying. The day of Hands is over. Machinery can do everything now and the Chinese might be left to die out as antediluvian survivals, like their buffaloes," she added, as he seemed unconvinced. "My dear Janie, have you never considered the delight of getting beyond railways, beyond steamers, among village industries into Ruskin's paradise? By Lethe's Stream!" he murmured, "though I am afraid there are telegraphs everywhere, even over the wildest passes." Lady Morten stared at him for a moment. From anyone else she would have thought this very silly. But from this time forward, she told all her intimates, "We are going to China to get rid of all the stress and strain of life in England." She did not feel the least strained or distressed herself. The composition of the yacht's crew was curiously unlike that of most yachts. The captain, a very brilliant naval officer, had thought he must give up his profession altogether because he could not stand the prolonged night exposure . . . he was a really admirable drawer of charts; . . . the first officer had tried to become a professional singer till his chest broke down with the English winter, while of the stewards one had been a lithographer, the other a photographer with artistic aspirations. Among the ship's company there was quite a galaxy of musical talent, also a hairdresser, and two men who described themselves quite simply as naturalists. The chef's aspirations were poetical. But it was only in his lighter moments that they were embodied in anything less piquant than a sauce. "But," he added, "the aspirations of my soul are for the most part expressed in my sauces. You can taste them there, can you not, milady? If you cannot I am sure monsieur can. He engaged me after eating one of my dinners, told me he wanted me to go among the yellow people, and the salary I might fix for myself. He wanted me to be content, and I am." This arrangement strikes Lady Morten as being very unbusinesslike, but her intention to reproach her brother for it is averted by the reflection "that the sauces were very good, and if his money was not spent in one way it would be in another."

Upon their arrival in Hong Kong Trevor Laurence is drawn against his will into the social gaieties, Lady Morten's impressions of the place are that England has done all it could be expected to do there "except teach the Chinese the laws of salutation and unbind their women's feet. It would be un-English to interfere with the domestic customs of another race," said Lady Morten. "A great many things are un-English which may yet be very desirable," replied her brother. ". . . Most of the Chinese are probably ashamed of foot binding; but the custom is too strong for them to do away with individually. They would probably be grateful for our help in the matter." "I daresay he may be right," said Lady Morten later to the governor's wife. "But that is what I am so afraid of about Trevor, that all his brilliant abilities will be wasted from his always fancying he knows what other people are wishing and feeling." "No one can tell what the Chinese are wishing and feeling. I have been here twenty years and have not the least idea," said a permanent official. Trevor Laurence is delighted with Macao. "If Hong Kong had delighted Lady Morten Macao delighted her brother, with its unexpected old-world picturesqueness and associations, its large crucifixes, little Portuguese soldiers, priests, duennas, and shall we add half-castes? . . . Women with Chinese features, but a sweetness of expression unknown in China. . . . He did not speculate about them or consider their future . . . he just enjoyed looking at them passing by, listening to the lapping of the waves upon the shore, wandering among the lovely crescent curve of the bay, or among the gardens by the sea, with their innumerable suggestions for vignettes, and spending long hours in the romantic melancholy of Camoens seat, where the poet is said to have composed the greater part of his world-fascinating epic." Trevor Laurence is enjoying the scene and the reflections it inspires when the sound of footsteps and a voice disturb him. "I think the Portuguese have thrown away all their opportunities here." He looks up and sees before him a tall overgrown girl. This is Bettina.

* "A Millionaire's Courtship." By Mrs. Archibald Little. (T. Fisher Unwin. 6s.)

### TRAVEL NOTES AND QUERIES.

By our Travel Correspondent.

A Happy and Inexpensive Holiday (Rustic).—You must tell me more before I can help you. What do you think constitutes a happy holiday? and what is your idea of inexpensive? Write me again exactly how much you can afford to spend, and if you prefer England or the Continent, seaside or inland places, for your holiday. I will then advise you for the best.

St. Malo or Cornwall (Lucy).—I do not think you could do better than act on the advice given to Mina—cost of journey, second class return from London, £1 19s. 2d. Your having no knowledge of French is of no consequence. Mrs. Dyott and her pleasant daughters are English. There are endless excursions to be made round the bay and inland. If you prefer North Cornwall, tell me, and I will give you addresses. You have plenty of money for the St. Malo trip.

In the Lauterbrunnen Valley (Ferien).—I do not think there is very much change in Isenfluh. Its position is very fine. I have only a record of one hotel, called Hôtel Pension, Yungfrau, with terms from 5 francs. My recollection of the place, seen three years ago, is of a quiet place. I can very seldom tell you how many rooms hotels have; and as to the food about which you inquire, I suppose it is the same as in most unpretending Swiss hotels—good and varied, but not elaborate. As to drains, do not make yourself unhappy about them, or Continental travelling becomes a horror. I know nothing of the drains at Isenfluh.

Three Weeks Abroad (Cigogne).—I see by your second card that the sum you have at your disposal is even less than I thought; it is not quite 3 francs 50 centimes a day, and it would be useless to attempt the Meuse on such terms, because, even if you could find quarters at that price you must reckon for washing and tips. The only two trips that will come comfortably within your means are those to St. Malo and to Bruges. See answer to (Pug) on April 28. I should counsel Bruges; there is so much to be seen all round, and so many interesting cities. You must not think of a tour, because frequent change of lodging greatly increases expense. If money held out well you might spend two or three nights in Brussels and return via Antwerp instead of Ostend. Tell me how I can help further.

Seaside near Ostend (An Old Londoner).—I think you may find what you want with Mme. Brunnen, Grande Hôtel, Knokke, a short distance from Ostend. Write to her on a prepaid foreign letter card and ask her terms for your party. It is a very good place for children, and you and your husband could make excursions to Bruges, Middelburg, etc. Journey via G.S.N.C. from Tower Bridge to Ostend, 10s. 6d. first return, 9s. second return. If you like Brittany try the Hôtel des Bains, Lancieux par Ploubaby, Côtes du Nord. Reached via St. Malo, the coast is fine, and I never think it too hot there. Many thanks for valuable address and information *re* the Jura. Holland is not to be thought of at your terms; it is an expensive country to travel in.

A Trip in Belgium (Wanderer).—You cannot do all you propose on the money you have; £7 each will only suffice for a fortnight's stay if you travel via Tower Bridge and Ostend. Antwerp is a longer voyage, and therefore more expensive. I do not know the terms of the Grimsby boats. Write for information, enclosing stamped envelope, to Messrs. Cook and Sons, Ludgate Circus, E.C. The cheapest place I can recommend at Antwerp is Hôtel du Commerce, 10 Rue de la Bourse. There are cheaper places among the Pensions, but you would not be taken for only two nights. At Brussels go to the Hôtel des Croisades or Hôtel du Rhin, 14 Rue de Brabant. At Ghent, Hôtel aux Armes de Zeelande, Marché aux Grains. All these about 7 francs per day. With regard to tips for one night, 1 franc each to waiter and the same to chambermaid and 50 centimes to the boots. For a longer period prices would rule less in proportion. It is not necessary to engage rooms beforehand in these cities. Ypres can be visited in one day from Bruges, but not from either of the cities you mention. My advice would be to go to Bruges and stay there, visiting (in day excursions) Ghent, Courtrai, Oudenarde, Ypres, and Ostend. French is not essential, but a slight knowledge of it makes a trip more enjoyable. Addresses at Bruges: Mlle. Boniface, 11 Quai au Miroir, terms from 5 francs; and Hôtel Panier d'Or, in the Market Place, from 6 francs.

### Notes and Queries.

**REGULATIONS.**

**The Editor is always willing to answer in this column, without any fee, all reasonable questions, as soon as possible.**

**But the following rules must be carefully observed.**

1. **Every communication must be accompanied by the name and address of the writer.**
2. **The question must always bear upon nursing, directly or indirectly.**

**If an answer is required by letter a fee of half-a-crown must be enclosed with the note containing the inquiry.**

*Education.*

(88) Can you tell me what standard of education is required from a candidate as nurse in the best hospitals?—*Inquirer.*

The standard is higher than formerly, but as long as you can understand the usual nursing text-books and are able to acquire the special knowledge necessary, we judge by your letter that you have nothing to fear.

*Age for Training.*

(89) Is 19-20 too young to commence training, and, if so, should my daughter go to a children's hospital to commence? —*Aspirant.*

Hospitals do not accept probationers under 23. The time spent at a children's hospital is not taken into consideration in general training.

*Nursing in France.*

(90) Can you help me as to nursing institutions in the South of France?—*Certificated.*

The Nice Nursing Institute, Villa Pilatte, Avenue Désambrois, and the Nursing Institute, Place de la Gare, Mentone. You had better apply now for next season; but there are few vacancies.

*Stewardesses.*

(91) How can I get to Australia or New Zealand free? Could I go as a stewardess?—*Eddie.*

There is no way that we know of securing a free passage, unless you were so fortunate as to take a patient out. No steamship company would take a stewardess for a single voyage.

*Massage at Cambridge.*

(92) Can you tell me where massage is taught in Cambridge?—*A Constant Reader.*

No doubt if you write to the Secretary of the Association of Trained Masseuses, 12 Buckingham Street, Strand, she will help you to find a teacher.

*Board and Lodging.*

(93) My patient gave me a week's payment instead of keeping me longer. Ought not I to have had board and lodging expenses too?—*Elizabeth.*

No. The law of master and servant provides notice or wages, not board wages.

*The Central Midwives Board Certificate.*

(94) What must I do to secure the Central Midwives Board certificate?—*Sister.*

"How to Become a Certicated Midwife," price 1s. 1d., post free, from The Scientific Press, 28 & 29 Southampton Street, Strand, London, W.C., gives full particulars.

*Home for Aged Person.*

(95) Can you tell me of a home for an old lady suffering from early stage of cancer?—*E. S. M. S.*

Write to the Sister in Charge, St. Peter's Home, Kilburn, N.W.

*Training.*

(96) I have had four years' training in an asylum. Will you advise me? Please give me addresses of public and private asylums. What should I get in a hospital? Is my certificate as good as that of the Medico-Psychological Association? *S. W. F.*

It would seem best for you to enter another asylum. You could not get one year's good training in a hospital without payment; you would have to train for three years. The Bristol City and County Asylum, Gloucester County Asylums, Wootton and Barnwood, and Somerset and Bath Asylum, Wells, are all within a short distance. We cannot give private addresses, but you will find some in "Medical Homes," price 7d., published by The Scientific Press. Your certificate is good enough, but try and get that of the Medico-Psychological Association as well.

*Compensation.*

(97) I accepted an engagement, and just before entering post I was told that I was not wanted. Can I claim a month's salary?—*Camberwell.*

Yes, if you have the engagement in writing and specified for a month's salary or month's warning. If not, consult a solicitor.

*Maternity Training.*

(98) Are there any maternity training schools in Liverpool that train free?—*Liverpool.*

We know of no such institutions.

*Tuberculosis.*

(99) Can you help me where to go for vapour baths and electricity? I am very helpless.—*L. F. C.*

Have you tried the Devonshire Hospital, Buxton? This might suit you. Write to the Secretary.

*Paris and Spain.*

(100) I have passed the Central Midwives Board examination; are there institutions in Spain or Paris I could join?—*Mrs. L. C.*

We know of no institutions in Spain. But you might write to English medical men in Spain and Paris and inquire as to probable success. There is a list of medical men practising abroad in "Medical Homes," price 7d., published by The Scientific Press, 28 Southampton Street, Strand, London, W.C.

*Nurses' Club.*

(101) Please give me address of Nurses' Club in London.—*F. P.*

You probably mean the Nurses' Co-operation, 8 New Cavendish Street. This institution employs nurses, who retain their fees less a percentage, and has a residential home.

*A Broken Engagement.*

(102) I was engaged by a lady, but a fortnight before the date she asked me to look for another case. I could not secure one. Can I claim a fortnight's board as well as half the fee?—*Nan.*

No; the half fee which you asked for is all that you can claim.

*Homes in Paris.*

(103) Can you tell me of a Nursing Home in Paris where English nurses are employed?

Write to some of the medical men mentioned in "Medical Homes," price 7d., published by The Scientific Press, 28 and 29 Southampton Street, Strand, London, W.C., they may be kind enough to advise you.

*Training.*

(104) Can you advise me where to apply for training? I am 5ft. 1in., and this has proved an obstacle so far.—*Petite.*

We can only advise you to persevere. Try provincial hospitals and you will probably find a vacancy, though your height is certainly a drawback.

*Naval.*

(105) Where must I apply for information to become a nurse at Naval Stations? Is any special training required?—*V. F.*

If you wish to join Queen Alexandra's Royal Naval Nursing Service you must write and ask for a form of application to the Director, General Medical Department of the Navy, Admiralty, 18 Victoria Street, S.W. Three years' training in a general hospital is required, and the candidate must be between 25 and 30.

*Nasal Catarrh.*

(106) Can you advise me where to go for advice about nasal catarrh? Please recommend a specialist or hospital.—*District Nurse.*

You will find a list of specialists in "Medical Homes," price 7d., The Scientific Press, 28 Southampton Street, Strand, London, W.C., or you might write and ask the Secretary of the Nose, Throat and Ear Hospital, Gray's Inn Road, W.C., to allow you to attend there and secure advice. State that you are a nurse.

*Midwifery Training.*

(107) Where can I get inexpensive but good midwifery training? I am 42 years old.—*Home.*

At the Maternity Charity and District Nurses' Home, Plaistow. This is recognised by the Central Midwives Board.

*Flat Foot.*

(108) I am flat footed; would this prevent me from becoming a nurse, and can you tell me how to cure myself?—*M. G.*

It would be a great mistake to attempt nursing under the present circumstances. You should consult a doctor, or go for advice to an orthopœdic or other hospital if you are not well off.

**Handbooks for Nurses.**

| | Post Free. |
|---|---|
| "How to Become a Nurse: How and Where to Train." | 2s. 4d. |
| "Nursing: its Theory and Practice." (Lewis.) ... | 3s. 6d. |
| "Nurses' Pronouncing Dictionary of Medical Terms." | 2s. 6d. |
| "Complete Handbook of Midwifery." (Watson.) ... | 6s. 4d. |
| "Preparation for Operation in Private Houses." ... | 0s. 6d. |

**Of all booksellers or of The Scientific Press, Limited, 28 & 29 Southampton Street, Strand, London, W.C.**

### For Reading to the Sick.

### THE WORSHIP OF HEAVEN.

INNUMEROUS choirs before the shining Throne
Their joyful anthems raise,
Till Heaven's glad halls are echoing with the tone
Of that great hymn of praise,
And all its host rejoices,
And all its blessed throng
Unite their myriad voices,
In one eternal song!

*J. M. Meyfart*, 1634.

The worship there shall be all praise. No prayer shall be there, for there shall be no sense of want. All is praise, for all is manifestation and light. All is praise, for all is triumph. All is praise, for all is blessedness and enjoyment. Whatever the feeling, praise, eternal praise, is the expression of it; from the breathing whisper of adoring love which flits through the prostrate ranks of the redeemed, to the full chorus of praise—the high, the universal shout of glory, and honour and blessing, to Him that sitteth upon the throne, and to the Lamb for ever.—*R. Watson.*

There is an unrest of pain, and there is a "rest not" of joy. It is the sweetest rest, to rest, and not to rest. For what is it but an unceasing, unwearying, unwearied rest, a river of joy which flows on in one peaceful fulness of bliss, without bound and without end? God giveth to the blessed, in their measure, to be like Himself. Here, to continue on in anything has weariness; because here is not our rest. There, upheld by God, the blessed behold the eternal truth without toil of thought; they, in spiritual bodies, move swiftly as the lightning without weariness; they love God above all things with everlasting love, and all besides with undivided love. They, in their degree, like Him, love none the less, because they love the others more. They love with a full undistracted soul, as God loves, at once, all whom He loves, with the fulness of His infinite love. And as our love, so will our praise be. What is praise, but to say how worthy of love is He Whom we love? But then we shall see without effort, praise without toil of seeking words in which to praise, with the whole unstrained power of our soul; unstrained, because sustained by God. Do we not find here, that if we would praise whom we deeply love, our words fail us? Is not our deepest praise to dwell in silent thought, gazing and feeling what is beyond our power to utter? And it is our deepest rest, so, entranced in love, to love without thought, or word, or motion, but in our inmost souls, to go forth out of ourselves, and dwell, without rest, on and in that which we love.

All, as it is most perfect here, is but a shadow of perfection there. Youth is but an image of everlasting freshness; beauty of form is but a faint picture of the brightness of immortal glory; the harmony of music, the most unearthly sound upon this earth, is but an echo of those angel choirs in which the redeemed shall fill up the perfect unison of the new heaven and the new earth. No joy of any sense shall be wanting there, but all shall be purified, heightened, glorified.—*E. B. P.*

## Figures and Tables

**Fig. 15. f1:**